# Peer mentoring for smoking cessation in public housing: A mixed-methods study

**DOI:** 10.3389/fpubh.2022.1052313

**Published:** 2023-01-05

**Authors:** Jummai Apata, Erica Goldman, Hamideh Taraji, Oluwatobi Samagbeyi, Shervin Assari, Payam Sheikhattari

**Affiliations:** ^1^Center for Urban Health Disparities Research and Innovation, Morgan State University, Baltimore, MD, United States; ^2^Resident Services Inc., Housing Authority of Baltimore City, Baltimore, MD, United States; ^3^Prevention Science Research Center, Morgan State University, Baltimore, MD, United States; ^4^Department of Urban Public Health, Charles R Drew University of Medicine and Science, Los Angeles, CA, United States; ^5^Department of Family Medicine, Charles R Drew University of Medicine and Science, Los Angeles, CA, United States; ^6^School of Community Health and Policy, Morgan State University, Baltimore, MD, United States

**Keywords:** smoking cessation, peer mentoring, community-based, health disparities, behavioral intervention

## Abstract

**Introduction:**

Tobacco use disproportionately affects low-income African American communities. The recent public housing smoke-free policy has increased the demand for effective smoking cessation services and programs in such settings.

**Methods:**

This mixed-method pilot study explored feasibility and potential impact of a peer-mentoring program for smoking cessation in a public housing unit. The quantitative study used a quasi-experimental design while qualitative data were collected *via* focus group discussions with peer mentors and participants. Three residents of the public housing complex were trained as peer mentors. Each peer mentor recruited up to 10 smokers in the residence and provided them individual support for 12 weeks. All participants were offered Nicotine Replacement Therapy (NRT). A follow-up investigation was conducted 3 months after completion of the 12-week intervention. At baseline and follow-up, the participants' smoking status was measured using self-report and was verified using exhaled carbon monoxide (eCO) monitoring.

**Results:**

The intervention group was composed of 30 current smokers who received the peer-mentoring intervention. The control group was composed of 14 individuals. Overall mean eCO levels dropped from 26 ppm (SD 19.0) at baseline to 12 (SD 6.0) at follow-up (*P* < 0.01). Participants who were enrolled in our program were more likely to have non-smoking eCO levels (<7 ppm) at follow-up (23.3%) compared to those who did not enroll (14.3%).

**Conclusion:**

Our program is feasible for low-income predominantly African American communities. Using peers as mentors may be helpful in providing services for hard-to-reach populations. Given the non-randomized design of our study, randomized trials are needed to test the efficacy of our program in the future.

## Introduction

After release of the first report of the Surgeon General's Advisory Committee on Smoking and Health in 1964, tobacco use began to decline. Subsequent interventions accelerated the decline, and today, the Centers for Disease Control and Prevention (CDC) reports that only 14.0% of adults in the U.S. smoke tobacco (1). Despite these overall gains from tobacco control programs, low income marginalized populations continue to be disproportionately harmed by smoking. The burden of tobacco is not evenly distributed; rather, shows large disparities by socioeconomic factors such as income, education, and race/ethnicity. Across the nation, smoking prevalence is inversely related to income and varies by racial and ethnic groups (1). In Maryland, Baltimore City has a higher prevalence of smoking and significantly greater health problems caused by smoking health disparities compared to the rest of the state. According to the Baltimore City Health Department, the 2014 smoking prevalence for Baltimore City was 33% while Maryland had a total smoking prevalence of 15.1% (2, 3).

Populations in the inner-city, including those living in assisted public housing, have some of the highest rates of tobacco use. Recipients of housing assistance in the U.S. are, by definition, socioeconomically disadvantaged (4–6). In 2017, more than one-third (33.6%) of adults receiving housing assistance were current smokers (7). The proportion of adults in U.S. public housing who reported excellent or good health was only 37.7%, compared to 48.7% of other low income people who did not live in public housing, and 66.8% of adults who were not poor (6).

The Department of Housing and Urban Development (HUD) implemented a Smoke-Free Housing policy on December 5, 2016, which became effective February 3, 2017. All Public Housing Agencies (PHAs) were to comply with the smoke-free policy within 18 months of the effective date. Managers and builders of these housing units were all expected to develop and implement plans to comply with the mandate. At the time of this study, fewer than 14% of public housing authorities in the U.S. had initiated smoke-free policies to limit smoking in residents' units (8). This mandate represented a valuable opportunity to test innovative smoking cessation services to help public housing authorities comply with the HUD initiative (9–11).

This study used a community-based participatory research (CBPR) approach, which is an effective model for reaching hard-to-reach populations with interventions based on behavior change. Because smoking is one of the biggest health threat that low income populations face, CBPR is especially important for reducing health disparities through smoking cessation. CBPR facilitates equitable engagement and participation of relevant community stakeholders in all aspects of the research to ensure shared ownership and more effective translation of the findings (12–16). CBPR-based interventions can be applied in diverse settings and facilitated by a diverse group of providers. The peer-facilitated CBPR approach is a promising model through which trained and experienced peers–an important group of stakeholders in CBPR–are engaged as educators, mentors, and sources for providing culturally relevant trainings and social support. Community-based peer-facilitated interventions have been shown to improve outcomes for interventions targeted at health-related behaviors (17–20), especially through providing smoking cessation services (20–22). Engaging peers brings valuable advantages for addressing health problems among vulnerable groups including those in mental health institutions, in drug recovery programs, and those who live in public housing (22, 23).

Given the role of feasibility studies to determine how programs can be more effectively implemented (24), we examined the feasibility and potential impact of a peer-mentoring smoking cessation program based in a public housing facility. The intervention adapted the structure, approach and curriculum of the Communities Engaged and Advocating for a Smoke-free Environment (CEASE), a well-established CBPR partnership in Baltimore Maryland (16, 25, 26). This intervention provided a valuable opportunity for a public housing complex to comply with the HUD initiative.

## Methods

### Study design and setting

This was a mixed method convergent parallel study design using quasi-experimental methods. The study was conducted in a federally subsidized public housing unit (Monument East apartments) in Baltimore City from 2016 to 2018. The unit was selected for the Peer Mentoring project because of its location in an underserved neighborhood and an anecdotal estimated smoking prevalence among its residents of 50%. In addition, Monument East had an existing relationship with community partners, provided accessibility to the target population (underserved residents of the apartments), and the facility's management were willing to support smoking cessation services for their residents.

#### Intervention

This intervention was guided by the Social support theory, a multi-faceted concept that has been applied to smoking cessation interventions with varying measures that assess the structure and function of social networks (27, 28). Social support encompasses any process through which social relationships can enhance health and wellbeing (29).

#### Mentor recruitment and training

Six former smokers who resided in Monument East Apartments were selected to participate as mentors in the program. These former smokers were trained on peer mentorship and smoking cessation, including motivational enhancement and relapse prevention, using the CEASE curriculum. They also received training on ethical considerations and the peer mentoring model for this project. At the end of their training, three participants were certified as peer mentors for smoking cessation. A toolbox of resources used in the CEASE program was provided to the mentors to supplement the support they provided to the participants based on individual needs. The mentors were trained and supported during the course of the intervention by experienced CEASE Peer Motivators (16, 25, 26).

#### Participant recruitment and training

Participants who were 18 years and older and were current smokers (defined as smoking at least three cigarettes per day in the past week) were recruited for the study through a baseline survey. Qualified participants who completed the baseline survey but did not enroll for the mentoring were regarded as controls. Participants were invited to take the survey through fliers posted in the building, word-of-mouth from mentors and other residents. Out of the 65 residents of the public housing unit who participated in the baseline survey, 44 were current smokers from whom 30 were enrolled and participated in the program.

The baseline survey was used to determine smoking rates among the residents and to recruit current smokers willing to join the program. Each peer mentor was instructed to reach out to approximately 10 participants who were qualified and willing to enroll in the program and establish a mentoring relationship preferably based on their existing relationships. Recruited participants received a single group counseling session facilitated by a CEASE Peer Motivator prior to entering into a “mentorship agreement” that included a quit-smoking plan and a follow-up plan. The Agreement described how mentors and participants would communicate and engage during the intervention. Options included face-to-face meetings, telephone calls, text messaging, emails, and any other modes of communication agreed upon by the parties. Mentors maintained logs of all communications with their participants and cessation outcomes. They kept track of the support they provided, their participants' experiences using NRT and participants' adherence to their quit plans.

#### NRT component

In addition, participants received a start-up dose of Nicotine replacement therapy (patch, gum or lozenges), depending on their level of nicotine dependency, which could be refilled if desired. While all participants received patch, receiving other forms of NRT was only based on request of the participant.

#### Incentive

Participants received cash incentives for participating in mentoring or counseling sessions ($10 per session) and follow-up interviews ($20). Participants received similar incentive at baseline and follow up regardless of their group membership. Thus, the incentive is not very likely to have biased the effect of participating in the intervention.

### Quantitative methodology

#### Timeline

The mentoring sessions lasted for 12 weeks. Twelve to 16 weeks after the mentoring phase, participants were contacted by mentors to complete a follow-up survey and determine their smoking status using an expired carbon monoxide monitoring test.

#### Data collection

Data were collected using paper-based surveys. The baseline survey captured information on demographics, physical and behavioral health, smoking history, barriers to quitting, stages of change and other variables. Participants completed progress forms during their interactions with the mentors which captured information on their adherence to their quit plan, motivators and barriers to quitting and aids for success. The follow-up questionnaires captured information on participants' smoking status and barriers to quitting.

#### Measures

##### Independent variable

Participants were categorized as “intervention-group” or “control-group” depending on whether they enrolled in the program or not. “Intervention-group” was coded as 1 and “control-group” was coded as 0.

##### Covariates

Sociodemographic characteristics included race, age, gender, employment status, marital status, and educational attainment.

##### Tobacco dependence

At baseline all participants were administered the Fagerström Nicotine Dependency Test to determine the intensity of their physical addiction to nicotine (30–32). Scores on the Fagerström Test potentially range from 0 (low) to 10 (high dependence).

##### Outcome

Quit and smoking status of each participant was ascertained during the peer-mentoring sessions, at the completion of the cessation program, and through the 12–16 week follow-up survey. Participants were categorized as “quit” or “didn't quit” based on self-reported smoking abstinence.

##### Confirmatory CO measurement

Verified by expired-air CO (eCO) levels. A level of 7 ppm was considered as “quit” while > 7 ppm was considered as “didn't quit”(33, 34). A carbon monoxide breath monitor was used to verify the smoking status of participants at every stage (34). Research has used a threshold of 7 ppm to indicate successful or unsuccessful quit. While eCO >7 ppm has been considered as “quit”, eCO > 7 ppm is considered as failure to quit (33). In our study, however, quit outcome was based on self-report and eCO measure was only confirmatory rather than being a criteria for successful quit.

#### Quantitative data analysis

Data was entered into EpiData version 3.1 (35) without personal identifiers and exported into STATA 14 (36) for cleaning and analysis. Descriptive univariate and bivariate analyses were conducted to review each variable and summarize demographic and baseline information by intervention arm. A bivariate analysis was done to compare baseline variables by intervention arm, using chi-square tests of independence for categorical variables (gender, race, education, marital status and employment status), and one-way Analysis of Variance (ANOVA) tests for continuous variables (age and Fagerström score). In estimating the study outcome, participants who did not complete the follow-up assessments were classified as “didn't quit”. This penalized imputation approach where participants with missing data were assumed to still be smokers has been applied as a method of conservatively estimating smoking cessation outcomes (37, 38).

#### Assessing feasibility

Indicators used to assess feasibility included acceptability, demand, implementation, practicality, and adaptation (24, 39, 40). These indicators were measured by counting the number of trainings provided, enrollment into the program, session attendance and retention, mode of interactions, NRT use, adherence to quit plan, support provided by mentor, and use of the resources in the toolbox.

#### Qualitative methodology

##### Intervention design

Qualitative inquiry using a phenomenological approach was applied to better understand the motivators of and barriers to smoking cessation (41). Using a convergent parallel design, findings from the quantitative and qualitative components of this study are integrated to examine factors that influence smoking cessation.

##### Sampling and recruitment

Participants were recruited through phone calls or word of mouth by site coordinators, Peer Motivators and peer mentors. Flyers were also posted in the relevant communities with contact information for recruitment. Focus group discussion (FGD) participants received financial incentives ($20) for their participation.

#### Measures

Interview guides were developed to capture participants' past experiences with smoking, quitting and the CEASE interventions; perceived barriers to quitting; expected aids for success; interactions with peer mentors (including perceived levels of social support provided) (42), strengths and weaknesses of the program; and recommendations for improving the program. Other measures included knowledge of smoking cessation resources and the public housing smoke-free policy. The interview guides for the peer mentors captured information on smoking and quitting history, participation motivators or barriers, experiences with the CEASE program and mentees, knowledge of the smoke-free policy and recommendations for future programs.

#### Data collection

Triangulation was achieved by collecting data from multiple sources. Three focus group discussions (FGDs) were conducted with a subset of program participants (*n* = 12) and one FGD was conducted with five peer mentors; three who continued and two who dropped out of the program. Focus group discussion moderator's guides were developed using semi-structured questions to probe for detailed information about smoking history, experiences with the intervention, motivators that helped and barriers that challenged efforts to quit, perceptions of the program's strengths and weaknesses.

#### Data analysis

The focus group discussions were audio recorded and transcribed into word documents. Transcripts were reviewed independently by two researchers and coded manually by reading through each transcript, extracting participants' responses and organizing them into relevant themes or subthemes in a tabular format. The integrity of the coding was verified through comparing and consolidating any disagreements between the emerging codes and themes generated through independent works of the two researchers. A convergent parallel design was used to integrate the results of the quantitative and qualitative components.

## Results

### Quantitative results

Table 1 shows the sociodemographic and baseline characteristics of intervention-group (*n* = 30) compared with the control group (*n* = 14). Men constituted 52.3% of overall participants, 52.8% were 55 years or older, and 95.4% were African American. Most participants (92.9%) were unemployed, 61.9% were single and 52.4% had graduated from high school. The mean Fagerström score was 4.6 (standard deviation = 2.2). All intervention group (100%) were unemployed, compared to 76.9% of the control group (*P* = 0.007). Of those who enrolled in the program, 66.7% had graduated from high school, compared to 16.7% of those who did not enroll (*P* = 0.004). Gender, age, race, marital status, and Fagerström scores were not significantly different in the group who enrolled, compared to those who did not.

**Table 1:** Sociodemographic and baseline characteristics of public housing participants.

**Variables**	**Not enrolled *n* = 14 (col %)**	**Enrolled *n =* 30 (col %)**	**All *n =* 44 (%)**	***P* value**
**Gender**				
Female	6 (42.9)	15 (50.0)	21 (47.7)	
Male	8 (57.1)	15 (50.0)	23 (52.3)	0.659
**Age**				
Mean (SD)	57 (11.2)	55 (9.1)	56 (9.8)	0.231
< 57 years	4 (33.3)	13 (54.2)	17 (47.2)	
≥ 57 years	8 (66.7)	11 (45.8)	19 (52.8)	0.238
**Race**				
African American	14 (100.0)	28 (93.3)	42 (95.4)	
White	0 (0.0)	2 (6.7)	2 (4.6)	0.323
Other				
**Employment**				
Full-time	3 (23.1)	0 (0.0)	3 (7.1)	
Part-time				
Unemployed	10 (76.9)	29 (100.0)	39 (92.9)	0.007
**Marital status**				
Single	9 (69.2)	17 (58.6)	26 (61.9)	
Married	1 (7.7)	8 (27.6)	9 (21.4)	
Other	3 (23.1)	4 (13.8)	7 (16.7)	0.319
**Education**				
Some high school or less	7 (58.3)	4 (13.3)	11 (26.2)	
Graduated high school/GED	2 (16.7)	20 (66.7)	22 (52.4)	
Some college or more	3 (25.0)	6 (20.0)	9 (21.4)	0.004
**Fagerström**				
Mean (SD)	5.1 (2.5)	4.3 (2.0)	4.6 (2.2)	0.8644
< 5	6 (42.9)	13 (44.8)	19 (44.2)	
≥ 5	8 (57.1)	16 (55.2)	24 (55.8)	0.903

A chi-square test of independence was performed for categorical variables (gender, race, employment, marital status, education). A one-way ANOVA test was performed for continuous variables (age and Fagerström score).

Selected quantitative outcomes are presented in Tables 2–**4**. The follow-up response rate was 80% (*n* = 24) for the intervention group and 71% (*n* = 10) for the control group, thus information on smoking status at three months post-intervention was only available for the 34 participants who completed the follow-up assessments. Sample sizes were not large enough to conduct multivariable or other more detailed analyses. The smoking cessation rate (CO levels <7 ppm) in the total population was 20.5% at follow-up. A chi-square test of independence compared smoking cessation rates between the intervention (23.3%) and control (14.3%) groups. This difference was not statistically significant (*P* = 0.488) (Table 2).

**Table 2:** Quit rates at follow-up among participants compared to non-participants.

**Variables**	**Outcomes**		***P* value**
**Arm** ^*^	**Quit**	**Didn't quit**	
	***n*** **(row %)**	***n*** **(row %)**	
Control (*n =* 14)	2 (14.3)	12 (85.7)	
Intervention (*n =* 30)	7 (23.3)	23 (76.7)	0.488

* Chi-square test of independence. Missing data at follow-up was classified as ‘didn't quit'.

Using a paired *t*-test, the mean CO level at baseline was compared with the mean CO level at follow-up for all participants. Within the total population, the mean follow-up CO level decreased by 14 ppm from the mean baseline CO level (*P* = 0.0009) (Table 3).

**Table 3:** Mean eCO levels at baseline and follow-up for all participants.

**Variables**	**Outcomes**		***P* values**
**CO** ^*^	**Baseline**	**Follow up**	
	**Mean (SD)**	**Mean (SD)**	
Overall CO	26 (19.0)	12 (6.0)	0.0009

* Paired t-test.

Two independent sample *t*-tests were performed to compare the difference in mean CO levels by enrollment status at baseline and at follow-up. At baseline, the enrolled residents had a higher mean CO level [27 (standard deviation = 18.6)] than those who had not enrolled [13 (standard deviation = 13.7)]. At follow-up, the difference in mean CO levels between the enrolled and non-enrolled residents narrowed [11 (standard deviation = 6.0) and 13 (standard deviation = 7.1) respectively], however, these differences were not statistically significant (Table 4). A chi-square test of independence comparing enrollment among residents who had CO breath tests at baseline with those who did not showed that having a CO breath test increased the likelihood of enrolling in the program; 90.3% of those who had a CO breath test enrolled in the program compared to 15.4% of those who did not have a CO breath test (*P* < 0.001) (Table 4).

**Table 4:** Mean eCO levels at baseline and follow-up by intervention arm.

**Variables**	**Outcomes**		***P* values**
**CO** ^*^	**Mean (SD)**	**Mean (SD)**	
Baseline CO	13 (13.7)	27 (18.6)	0.2777
Follow-up CO	13 (7.1)	11 (6.0)	0.5366
CO^**^	n (row %)	n (row %)	
Had baseline CO testing	3 (9.7)	28 (90.3)	
No baseline CO testing	11 (84.6)	2 (15.4)	P < 0.001

* independent t-test,

** Chi-square test of independence.

An evaluation of the feasibility of the peer mentoring program based on selected indicators (acceptability, demand, implementation, practicality, and adaptation) is presented in Table 5. Six mentors were trained and three continued with the program while three others could not continue for personal reasons. Each of the 30 enrolled participants was expected to attend a maximum of seven sessions over the course of three months. Outcomes from a total of 197 interactions between participants and their mentors are presented. They show that 56.9% of participants completed four sessions out of the seven; 98.0%of these sessions were held as face-to-face meetings while 2% were through other means such as phone calls and text messages. Use of NRT was reported in 49.8% of these interactions from whom 53.8% found NRT to be helpful. Challenges with the NRT were reported in 3.6% of the interactions and 17.8% had challenges sticking to their quit plan. Support was provided to the participants by their mentors based on the four A's of dealing with challenges to quitting. Most interactions involved support for finding **A**lternatives to smoking (40.6%), and **A**voiding situations that could trigger smoking (40.1%). Other support included alternative **A**ctivities in which to engage (17.3%) and **A**ltering routine habits (14.7%) to curb the cravings. Based on options used from the toolbox provided to mentors the most common topics discussed included relapse prevention, withdrawal, healthy relationships and managing stress.

**Table 5:** Assessing feasibility of implementation.

**Indicator**	**Achievements**	**Remarks**
**Acceptability and demand**		
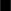 Trainings	Six mentors trained, 3 (50%) retained	
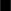 Enrollment	30 current smokers (68.2%) enrolled; 90.3% of current smokers who had CO testing enrolled; 15.4% of those who had no CO test enrolled	
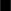 Attendance and retention	43.1% attended up to 5 sessions; 56.9% attended up to 4 sessions	
**Implementation and practicality**		
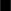 Mode of interaction	98.0% face-to-face	
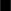 NRT; 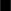 Use of NRT; 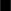 Type of NRT; 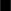 Benefit of NRT; 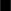 Challenges with NRT use	49.8% used NRT; 23.4% used Gum, 21.3% used Patches, 14.2% used Lozenges; 53.8% of the users found NRT helpful; 3.6% of the users had problems with NRT	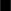 NRT problems: chest pain, shortness of breath, did not like it
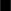 Quit plan 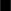 Adherence to quit plan 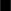 Challenges with adhering to quit plan 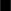 Changes to quit plan	17.8% had challenges sticking to quit plan	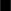 Mentee: Doing good, ok, coming along 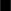 Trying to slow down, cutting back 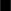 Mentor: Doing good, making progress, not doing good, trying, continue to show up at meetings, needs to slow down 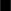 Challenges: strong urges, caffeine intake, facing stressful events, urge to eat more, did not have money for cigarettes, around people who smoke, health and housing issues 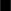 Changes: Share with the group, try patches and gum more, no change, stick to plan
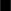 Support provided by Mentor based on the 4 A's	Avoid: 40.1% Alter: 14.7% Alternative: 40.6% Activities: 17.3%	
**Adaptation**		
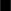 Use of toolbox resources	Relapse prevention Withdrawal Healthy relationships Stress	

### Qualitative results

Table 6 presents our findings from the qualitative assessment of the public housing peer mentoring program. Themes and subthemes from three FGDs held with a total of 12 participants. The themes describe participants' characteristics, their experiences with the program and their recommendations for future programs. FGD participants' ages ranged from 50-67 years; six of them were men, and six were women.

**Table 6:** Qualitative findings from public housing peer mentoring project.

**Themes/Subthemes**	**Participants**		**Mentors**	
	**Findings**	**Quotes**	**Findings**	**Quotes**
**Participants' characteristics**				
• Demographics •Smoking history • Enrollment motivators • Perception of policy	• Six males, six females, mostly seniors, low income • Most participants started smoking at a young age (13–18 years); many were influenced by their peers, family and friends to start smoking; all of them knew about the negative health effects of smoking • Some participants were motivated by the financial incentives; some were motivated by health concerns. • Most participants were aware of the HUD smoke-free policy, most of them did not support the policy.	“*Well I started at the age of 13. It's something I wanted to do and something that was decided by me, nobody else or anything like that, it was just something that I wanted to do. My father was a smoker...”* “*It interferes with* *... just general overall health.”* “*It was, like I said, the money first. And I was thinking when I get into it I might change my mind. I might feel different about it and that's how it worked for me.”* “*I wasn't worried about the money part, just my health. And I noticed that I had more trouble breathing since I smoked them couple of cigarettes yesterday.”* “*I don't like it.” “When you go in your apartment and shut the door you should be able to smoke if you want to.”*	• Two males, one female, ages 50–67 years, low income • They started smoking between ages 16 to 18 • They were encouraged by the Resident Services Coordinator to enroll They were all aware of the HUD smoke-free policy and most of them did not support the policy.	“*It all started off when I was drinking wine when I was sixteen'* “*We enrolled as soon as you told us...”* “*Just gotta' be 25 feet away from the building... and they get these special devices so they can detect who was smoking in their apartments.”* “*Nobody can tell you what to do in your own place.”*

#### Smoking history

Most participants initiated smoking at a young age. Some participants were influenced by peers or family members to start smoking while others initiated smoking due to life stressors.

#### Enrollment motivators

The participants heard about the CEASE program mostly *via* word of mouth, and also through the site coordinator or flyers posted in the building.

#### Motivation to participate in the program

The motivation to join the program ranged from health reasons to family. For some of them, the incentives motivated them to participate and remain in the program. Some residents who did not participate cited not having enough time as a reason for not participating.

#### Experience with the peer mentoring program

Participants cut down the number of cigarettes smoked or stopped and started again. Some aids to quitting as mentioned by participants included the education they received in the CEASE classes, the peer support they received and the NRT provided. For some participants, they mentioned that NRT was not very helpful for them. Some barriers for quitting identified by participants included low self-efficacy and being around others who smoke. In terms of the program, most participants said that they found the curriculum helpful and educative. They also found the visual aids about harmful components of cigarettes helpful. A few participants noted an improvement in their health due to the change in their smoking behavior during the program. Almost all participants agreed that meeting in a group helped them stay involved and feel less alone.

#### Perception of the smoke-free policy

In terms of the smoke-free policy, most residents (intervention and control) were aware of the public housing smoke-free policy. A few of them were in support of it but most of them had certain misgivings about the policy.

Table 7 also presents findings from the qualitative assessment of mentors who were trained for the public housing peer mentoring program. At the end of the training, three trained mentors decided not to continue with the program due to other conflicting time schedules. Themes and subthemes from three FGDs held with a total number of six mentors (three who continued in the program and three who were unable to continue) are presented in this table. The themes describe participants' characteristics, their experiences with the program and their recommendations for future programs.

**Table 7:** Qualitative findings from public housing peer mentoring project.

**Themes/Subthemes**	**Participants**		**Mentors**	
	**Findings**	**Quotes**	**Findings**	**Quotes**
**Experiences**				
• What they liked • Perceived impact • Logistics • Areas for improvement	• Most participants liked the shared experiences in group sessions. • Many said the program had led them to cut back on the number of cigarettes they smoked. • Some thought their Mentors could benefit from more training. • Some felt that the program should go on longer.	“*Really, I like the group.”* “*In a group setting, you can actually learn from other people.”* “*One thing, I did slow up on smoking from like a pack and a half to around to a half a pack.”* “*I had a good Mentor. but yeah, I think if they were trained a little more to me maybe well it depends on what people want to do* “*Training, pamphlets, discussing the questions are all asking you to get to each person's opinion.”*	• All of them enjoyed the experience and expressed willingness to serve as Peer Mentors for similar programs. • They felt that the training they received increased their capacity to be Mentors. • Some Mentors experienced challenges with their mentees. • Some mentees thought that participants would benefit from more information before recruitment. • Some participants wanted the program to go on longer.	“*It was fun to me, once I made up my mind that's what I was gonna' do.”* “*Yeah, the training was fine.”* “*Right, right, they will always get on your nerves. They always got problems. You try to deal with ‘em; some of em always got problems.”* “*Explain it to ‘em better.”* “*Some of ‘em felt like it wa'nit long enough.”*
Recommendations	Common suggestions included advertising the program more, providing gift cards, more refreshments, and using visual aids.	“*Give gift cards instead of cash. Target or some food place...”* “*They can add advertisement.”* “*A lot of people can't read or comprehend but they can see the pictures.”*	Participants suggested increasing monetary incentives and refreshments	“*They normally come to things that feed ‘em first. You show ‘em some food and you might get a whole lot of cooperation out of ‘em.”* “*Food and money.”*

#### Experience with the peer mentoring program

All mentors got to know about the program through the resident service coordinator who encouraged them to participate as mentors due to their non-smoking status. Most peer mentors felt that they would need further training to build their capacity as mentors. Overall, they enjoyed the mentoring experience and said they were willing function in that capacity for future programs. Some mentors however expressed having some challenges with the process of supporting the participants. They felt that they needed more time to work with mentees.

#### Perception of the smoke-free policy

The mentors were all aware of the smoke-free policy, however, some of them had reservations about the smoke-free policy being rolled out in their building.

#### Recommendations and areas for improvement

Most mentors recommended maintaining monetary incentives and providing refreshments to motivate participants to remain in the program. They also recommended reducing the amount of paperwork involved in implementing the program.

## Discussion

Our pilot study applied a mixed methods design to explore the feasibility and potential efficacy of a peer-mentoring program in public housing in response to HUD's announcement of the smoke-free housing initiative. We found preliminary results suggestive of the potential efficacy of peer mentoring for tobacco cessation. We discuss some of our main quantitative and qualitative findings here but given the limitations of this study such as small sample size and quasi-experimental design with lack of randomization, future research is needed to further confirm these results.

In this pilot study which was conducted in a public housing unit in Baltimore City, residents were recruited and offered smoking cessation classes led by peer mentors who were themselves former smokers. Because the peer mentors lived in the same complex, they were able to have frequent and personal interactions with the participants outside the class setting. The participants could directly observe the smoke-free lifestyle of their mentors and model their healthy behaviors. Participants could easily contact their mentors when facing any challenges. Mentoring has been documented in prior research to be beneficial for addressing a wide range of behavioral and health problems including smoking (43, 44).

More than 23% of the intervention group had nonsmoking eCO levels at three-month follow-up compared to 14.3% of the control group. However, due to the small sample size, this difference did not reach statistical significance. Another notable finding was an overall decrease in the mean eCO levels for all participants (enrollees and non-enrollees) from 26 ppm at baseline to 12 ppm at follow-up (SD = 19.0 and 6.0 respectively). It is interesting that 14.3% of non-enrollees also achieved smoking cessation eCO levels, and that all participants achieved a decrease in mean eCO levels. While contamination of the control group (non-enrollees) due to diffusion of information from enrollees cannot be ruled out, other factors such as the prevalence of unassisted quitting in the general population might be considered. Enrollees and non-enrollees all lived in the same complex and in some cases were housemates or spouses. The information provided to enrollees and their lifestyle modifications resulting from the mentoring sessions could have been adopted by non-enrollees who knew them or lived with them. This could be considered a form of contamination of a control group (45). These results could have important implications for smoking cessation efforts in public housing and other residential complexes where lifestyle and behavioral changes introduced by a small group of persons diffuse to other residents causing unintended but positive health outcomes (46).

Certain indicators were utilized to determine the feasibility of this intervention and to examine its scalability. Almost 70% of surveyed residents who were current smokers enrolled in the mentoring program, suggesting the attractiveness of cessation services in this group even though prior research has shown that underserved populations have lower intentions to quit (47). The provision of cash incentives to participants could also have increased the interest in participation and engagement in the program. About 60% of residents who enrolled in the program attended at least four of the seven mentoring sessions offered. Considering the high risk of drop-out in this type of population (13, 48), this was a promising outcome that might have resulted from the convenience of the intervention being located in the same building where the participants resided. A summary of 197 interactions that the 30 mentees had with their mentors over the three-month period of the study revealed useful information about NRT use in this population. NRT use was reported in only about half of the sessions. The most commonly used form of NRT was gum (23.4%); the least used form was lozenges (14.2%). NRT use was reported to be helpful in 53.8% of interactions with those who used the products. However, 3.6% of the participants reported problems including chest pain and shortness of breath after using NRT, or that they just “did not like it.” Studies show that although adverse effects of NRT are rare, some people do experience discomfort with all forms of NRT. According to the authors of these studies, the adverse effects according are not sufficient to recommend discontinuation of NRT (49, 50).

Results showed that about 18% of participants had difficulty with adhering to their quit plans. Reported challenges included facing stressful events, having strong urges, and dealing with triggers such as caffeine. Barriers to quitting that have been well documented in prior research include being around people who smoke, having strong cravings and stressful events (51, 52). In describing the barriers to quitting, one participant said “When I'm around people that start smoking, the Jones start to kick in, the urge starts coming in and if I don't leave I am going to ask for one…” Most participants however, were motivated to at least cut back. They cited health problems, not having money for cigarettes and the peer support they received as factors that motivated them to adhere to their quit plans (53). One participant stated “I needed the program myself ‘cause I was in bad shape at that time with my lungs”.

Mentors supported participants by using the four A's for dealing with barriers to quitting. Of the 197 interactions, the most support was provided on “Alternatives” to smoking (40.6%), and “Avoiding” certain places or people (40.1%). The most discussed resources from the toolbox provided to mentors were relapse prevention, withdrawal, healthy relationships and stress (54–56).

Smoke-free public housing policies are intended to reduce secondhand smoke exposure for residents. Research shows that such policies also help to reduce smoking rates (57). Residents of public housing have been found to have higher rates of smoking compared to the general population. Public housing residents are typically of low socioeconomic status and tend to have a myriad of social and environmental factors that increase their chances of engaging in unhealthy behaviors such as smoking (4–7). They also experience poorer health compared to the general population, which interacts in a cyclical way to further increase their everyday stress and worsen their health and health behaviors (6).

Smoke-free initiatives are important steps in protecting the health of poor and underserved public housing residents, including smokers and nonsmokers (58–61). The reduction in secondhand smoke exposure for infants, children, seniors and persons with disabilities in public housing could be pivotal in addressing long-standing tobacco-related health disparities (58–61). The initiative is also expected to result in substantial cost savings in healthcare expenditures (62) and even in the costs of cleaning units for new tenants.

Determining the feasibility of this program in public housing is essential because of the documented challenges of involving these populations in smoking cessation research and interventions. Residents of public housing have traditionally been hard-to-reach populations with competing socio-environmental pressures that make them less willing to enroll (63) and participate in such programs (64). Residents may view interventions directed at changing their health behaviors as paternalistic, thereby creating mistrust and limiting their participation (65). Interventions that are not culturally specific or tailored to the target population may therefore not succeed (48). A study by Geller, Rees and Brooks (2016) showed that minorities in public housing reported a lower use of NRT than their White counterparts (58). As discussed previously, recruitment, class attendance and retention have been challenging for this population. That is why these specific indicators were assessed in the current study.

One of our lessons learned was that the location of the smoking cessation services in the same complex where participants lived may have contributed to the success of our program. Delivery of services by peers who resided in the same building and had lived through similar experiences was reported to be instrumental in establishing trust and building mentoring relationships with the participants. Additionally, providing cash incentives likely encouraged participation and engagement in the program. Lastly, strong partnership with involvement of diverse community stakeholders contributed to creation of a shared sense of ownership of the program and its successful implementation.

History of CEASE program, as a long-standing community-academic partnership, was critical to the overall successful implementation of this intervention. The engagement of all stakeholders in every step of this research promoted capacity-building of academic researchers and community leaders at every level. The shared decision-making process encouraged a feeling of ownership by all stakeholders that is a crucial element for sustainability. The use of feedback and the incorporation of lessons learned from each step of the process guided the development of the intervention in an iterative, progressive manner and was a unique strength of this study. The partnership was adept at balancing action with research with the overall mindset of doing research “with” the community and not “on” them.

A mixed method approach with triangulation was employed to improve the standard and quality of data. Examining the interventions using both quantitative and qualitative approaches provided ample depth about the outcomes. The strength of the CEASE partnership came from expertise in diverse fields and was valuable for addressing the limitations described at each stage of the interventions.

This study had a few limitations. Due to the small sample size, some of the differences were not statistically significant. We however included a qualitative component to provide a more in-depth understanding of the study results. In addition, some mentors who received the training were not able to continue for personal reasons. Several mentees were therefore reassigned to other mentors to ensure continuity. Other limitations included a quasi-experimental design and lack of randomization. We were however able to follow up smokers who did not enroll into the program as a control group. The control and intervention groups may not have been completely comparable as seen by the differences in willingness to participate in the program which was an enrollment criteria. We, however, included a variable in the baseline survey to measure the respondents' intention to quit based on Prochaska's stages of change theory (66). This variable which helped us determine the level of motivation to quit smoking, was not significantly different for the two groups at baseline. We also offered CO monitoring as a motivational tool to all respondents of the survey at baseline before they decided whether or not to enroll. Given all these limitations and given our design, we cannot attribute all positive results to the intervention without any consideration that there might be other causes for the findings. For example there were some baseline differences between those who took part in the intervention and those who didn't. Similarly, some people who didn't receive the intervention quit smoking, and we cannot attribute this finding to contamination.

Despite the limitations of this pilot study, our mixed-methods results introduce peer-mentoring smoking cessation interventions as an innovative strategy for high-need settings such as predominantly African American low-income public housing residential complexes. Although more research is needed, such an approach may create an important opportunity for quitting smoking through training local peers and empowering public housing residents so that they can get more involved in serving their own communities. Our intervention was successful because our tobacco cessation curriculum was culturally adapted to this setting. Similar cultural adaptation is needed for other diverse groups and vulnerable populations. For example, young people, who are targeted by tobacco companies, could benefit from an adapted version of this intervention (i.e., a web version of the program). Implementation and evaluation projects should test the effects of our peer mentoring model in other similar settings.

## Data availability statement

Due to privacy and ethical concerns, neither the data nor the source of the data can be made available.

## Ethics statement

This study involving human participants was reviewed and approved by Morgan State University Institutional Review Board. The participants provided their written informed consent to participate in this study.

## Author contributions

Material preparation, data collection, and analysis were performed by JA, HT, EG, and OS. The first draft of the manuscript was written by JA and SA. PS reviewed and revised following versions of the manuscript. All other authors reviewed and commented on previous versions of the manuscript. All authors contributed to the study conception and design and read and approved the final manuscript.
